# Liposomal delivery of azithromycin enhances its immunotherapeutic efficacy and reduces toxicity in myocardial infarction

**DOI:** 10.1038/s41598-020-73593-0

**Published:** 2020-10-06

**Authors:** Ahmed Al-Darraji, Renée R. Donahue, Himi Tripathi, Hsuan Peng, Bryana M. Levitan, Lakshman Chelvarajan, Dalia Haydar, Erhe Gao, David Henson, John C. Gensel, David J. Feola, Vincent J. Venditto, Ahmed Abdel-Latif

**Affiliations:** 1 Gill Heart and Vascular Institute and Division of Cardiovascular Medicine, University of Kentucky, Lexington, KY USA; 2 College of Pharmacy, University of Kentucky, Lexington, KY USA; 3 The Center for Translational Medicine, Temple University School of Medicine, Philadelphia, PA USA; 4 Spinal Cord and Brain Injury Research Center, Department of Physiology, College of Medicine University of Kentucky, Lexington, USA; 5 Division of Cardiology, University of Kentucky and the Lexington VAMC, 741 S. Limestone Street, BBSRB, Room 349, Lexington, KY 40536-0509 USA

**Keywords:** Cardiovascular biology, Cardiology

## Abstract

A growing body of evidence shows that altering the inflammatory response by alternative macrophage polarization is protective against complications related to acute myocardial infarction (MI). We have previously shown that oral azithromycin (AZM), initiated prior to MI, reduces inflammation and its negative sequelae on the myocardium. Here, we investigated the immunomodulatory role of a liposomal AZM formulation (L-AZM) in a clinically relevant model to enhance its therapeutic potency and avoid off-target effects. L-AZM (40 or 10 mg/kg, IV) was administered immediately post-MI and compared to free AZM (F-AZM). L-AZM reduced cardiac toxicity and associated mortality by 50% in mice. We observed a significant shift favoring reparatory/anti-inflammatory macrophages with L-AZM formulation. L-AZM use resulted in a remarkable decrease in cardiac inflammatory neutrophils and the infiltration of inflammatory monocytes. Immune cell modulation was associated with the downregulation of pro-inflammatory genes and the upregulation of anti-inflammatory genes. The immunomodulatory effects of L-AZM were associated with a reduction in cardiac cell death and scar size as well as enhanced angiogenesis. Overall, L-AZM use enhanced cardiac recovery and survival after MI. Importantly, L-AZM was protective from F-AZM cardiac off-target effects. We demonstrate that the liposomal formulation of AZM enhances the drug’s efficacy and safety in an animal model of acute myocardial injury. This is the first study to establish the immunomodulatory properties of liposomal AZM formulations. Our findings strongly support clinical trials using L-AZM as a novel and clinically relevant therapeutic target to improve cardiac recovery and reduce heart failure post-MI in humans.

## Introduction

Myocardial infarction (MI) remains a primary cause of morbidity and mortality globally^1^. Despite the significant improvement in post-MI outcomes over the past two decades, millions of patients suffer adverse cardiac remodeling and heart failure^1,2^. Cardiomyocyte death and extracellular matrix (ECM) breakdown at the area of cardiac injury induce a potent and poorly controlled inflammatory response^3^, interfering with healing and scar formation processes^4–6^. Innate immune phagocytes such as neutrophils, monocytes, and macrophages are key players in this response^7^. Neutrophils are the first line of immune cells arriving in the myocardium after injury and they play an important role in the development of adverse remodeling as well as healing of the myocardium^8,9^. Neutrophils are then followed by two waves of monocytes, pro- and anti-inflammatory monocytes which convert to tissue macrophages that play a crucial role in the healing myocardium^10^.

Macrophages are essential in organizing the early post-MI inflammatory and subsequent reparative phases. They are generally classified into pro-inflammatory/classically activated/M1-like and anti-inflammatory/alternatively activated/reparative/M2-like macrophages based on gene profile and function^11,12^. Pro-inflammatory macrophages are dominant 1–3 days post-MI and mediate the inflammatory process^11,13^. Following this early inflammatory phase, reparative macrophages are the most prevalent and are characterized by high expression levels of interleukin (IL)-10, IL-1ra, and decoy type II receptors^11,14,15^. As a result of the production of these mediators, M2-like macrophages play a central role in inflammation resolution, thus promoting tissue healing and angiogenesis^16^. There is a growing body of evidence suggesting that alternative polarization of macrophages is protective against the early development of ischemic lesion and subsequent adverse cardiac remodeling^17–21^. Previous research from our lab suggests that azithromycin (AZM), a commonly used antibiotic with immunomodulatory effects, alters post-MI inflammation through repolarization of macrophages to a reparative phenotype. These immunomodulatory effects were associated with protection against adverse cardiac remodeling and heart failure^22^. However, these findings have limited clinical translation given the pre-MI initiation of therapy and the relatively high dose (160 mg/kg/day, orally), which were dictated by our non-targeted delivery of AZM^22^. Additionally, studies show that AZM produces negative inotropic and chronotropic effects and delays ventricular repolarization^23^. These hemodynamic effects of AZM are likely mediated by a Ca^2+^ channel-independent pathway and delayed rectifier K^+^ current^24^. Additionally, the cardiac action potential is much shorter in mice compared to humans, indicating potentially more arrhythmogenic potential in humans compared to murine models^25^. The goal of this translational study is to determine the efficacy and safety of encapsulated AZM as immunotherapeutic agent for MI, using a clinically relevant experimental design.

Several therapeutic agents have been tested to alleviate cardiac remodeling post-MI through systemic administration; however, they suffer limited efficacy due to poor distribution into the heart or dose-limiting adverse effects^26^. The clinical use of anti-inflammatory agents for post-MI inflammation have been hampered by poor cardiac bioavailability, systemic side effects and dose limiting cardiotoxicity. The increased vascular permeability post-MI facilitates the accumulation of nanocarriers such as liposomes in the injured myocardium^27^. Liposomes are formed of one or more phospholipid bilayers surrounded by an aqueous core, which can hold both lipophilic and hydrophilic compounds. Given their biocompatibility and ease of use, they have rapidly become an attractive drug delivery system^28^. Liposomes reduce the ED50 of the cytotoxic drug doxorubicin while promoting anti-tumor efficacy^29^. Doxorubicin liposomes reach steady state faster and with fewer doses due to intensified localization to targeted organs^30^. Theoretically, liposomes are highly effective carriers after MI, achieving higher drug concentration at sites of cardiac injury due to increased vascular permeability^27,31,32^. Indeed, liposomal delivery formulations are effective drug delivery strategies for phagocyte-targeted therapy, producing lower immunogenic reaction, higher biocompatibility and specificity, and greater drug stability^33^. However, there is no data on the effectiveness of liposomal formulations in drug delivery after MI. Specifically, the ability to enhance AZM targeted drug delivery to inflammatory phagosomes has not been previously tested.

This study is designed to achieve targeted AZM delivery post-MI and improve its immunomodulatory benefits while minimizing dose and off-target effects. Using non-PEGylated liposomes as a delivery tool (L-AZM), we were able to significantly improve the safety of AZM therapy after MI by reducing cardiotoxicity. Additionally, liposomal encapsulation enhances the immunomodulatory effects of AZM on inflammatory phagocytes, while providing cardioprotection through decreased cardiomyocyte drug exposure. This data suggests a safe and clinically relevant pathway for targeted drug delivery to the post-MI myocardium in patients.

## Materials and method

Full details on the materials and methods are included in the supplemental data.

### Study design

**Figure 1:**
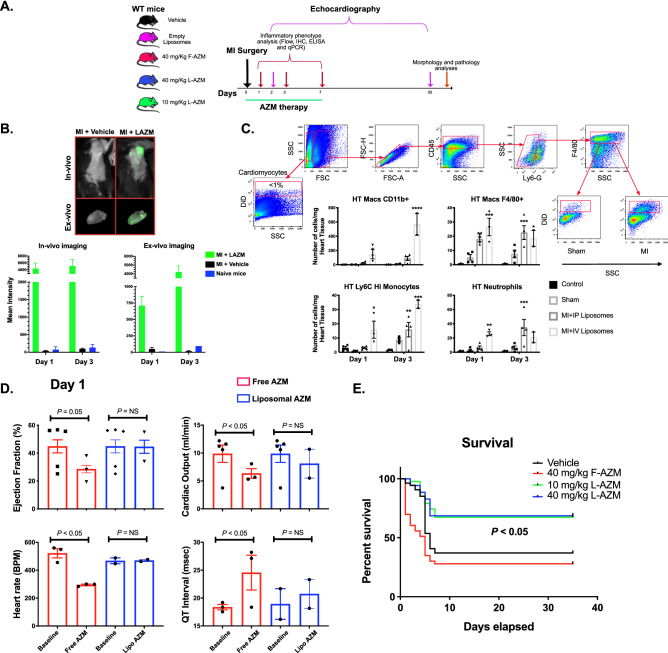
Liposomal azithromycin formulation provides a safer and more specific delivery tool after myocardial infarction. (**A**) In vivo experimental design targeting myocardium with F-AZM or L-AZM after infarction. MI was induced by permanent ligation of the left anterior descending coronary artery. (**B**) Maestro images of hearts (ex vivo) after DiD labeled L-AZM injection, vehicle 1 and 3 days after infarction. Lower panel shows quantitative assessment of signal intensity in the heart using ex-vivo imaging showing an increase in signal intensity in MI + LAZM hearts in comparison to vehicle and empty liposomes control (1–3 animals/time point). (**C**) Flow cytometry analysis of DiD labeled L-AZM at days 1 and 3 post-MI showing liposomal accumulation in cardiac immune cells such as macrophages after IP or IV administration, with more significant accumulation effect after IV administration. (**D**) Hemodynamic effects of free and liposomal AZM. Pre- and immediately post-administration measurements of ejection fraction, cardiac output, heart rate, and QT interval after retro-orbital administration of 40 mg/kg of liposomal or free AZM. Mice were examined 1 day after MI. Data shows that the liposomal formulation of AZM is protective against chronotropic and inotropic changes induced by free AZM (N = 2–5 animals/group). (**E**) Survival curves of free/liposomal AZM and vehicle-treated mice to 35 days post-MI. Mortality in 40 mg/kg IV AZM group occurs during the drug administration due to severe changes in cardiac conduction and hemodynamic parameters (**C**,**D** n = 3 animals/gr oup/time point. **E** Vehicle group, n = 34, 40 mg/kg IV AZM group, n = 33, 40 mg/kg LAZM group, n = 35, and 10 mg/kg LAZM group, n = 31, *P < 0.05, **P < 0.01, and ***P < 0.001, and ****P < 0.0001 compared to sham). Data presented as mean ± SEM. IV, intravenous; IP, intraperitoneal; F-AZM, azithromycin; L-AZM, liposomal azithromycin; Macs, macrophages; Control, mice did not receive liposomes.

C57BL/6 male mice (Jackson Laboratory, Bar Harbor, ME), age 6–8 weeks, were treated with liposomal or free AZM (40 mg/kg/day) or vehicle (PBS or empty liposomes) using retro-orbital (intravenous-IV) or intraperitoneal (IP) injection daily, starting immediately after MI or sham surgery and continuing for 7 days (Fig. 1A). We added an additional group examining the efficacy of lower dose L-AZM (10 mg/kg/day) to assess the effectiveness of liposomal formulation in reducing the effective dose of AZM. This administration strategy was chosen to mimic commonly prescribed AZM regimens in patients being treated for infection^34^ and provides effective distribution and tissue accumulation^35^. Treatment was continued for 7 days to encompass the duration of the post-MI inflammatory phase^36^. All surviving animals were followed for up to 35 days for survival, cardiac functional recovery and scar formation. All procedures were conducted under the approval of the University of Kentucky IACUC in accordance with the NIH Guide for the Care and Use of Laboratory Animals (DHHS publication No.[NIH] 85–23, rev. 1996).

### Murine model of myocardial infarction (MI)

MI was induced using the permanent left anterior descending coronary artery (LAD) occlusion method as previously published^22^. Sham surgery consisted of the same procedure without LAD ligation.

### Preparation of liposomes

Liposomal formulations were prepared according to a previously established protocol^37,38^, using the thin film hydration method with equal molar ratios of distearyl phosphatidylcholine (DSPC), distearyl phosphatidylglycerol (DSPG) and cholesterol (Avanti polar lipids). AZM was included at 10 and 30 mol% based on phospholipid content. Formulations containing fluorophores were prepared in the same manner with 0.5 mol% of the lipophilic dye, 1,1′-dioctadecyl-3,3,3′,3′-tetramethylindodicarbocyanine (DiD). Liposome size, polydispersity and stability over time were determined by dynamic light scatter testing.

### Flow cytometry

#### Peripheral blood (PB)

Cell phenotype, in addition to gene analyses, of monocytes and neutrophils was examined in peripheral blood as previously described^22^. Cells were lysed then washed twice in staining buffer to remove any residual lysis buffer. Cells were incubated with conjugated primary antibodies against Ly6C/G, F4/80, CD11b, CD45, and CD115 (Biolegend) for 30 min on ice. Following incubation, cells were washed twice with staining buffer. Monocytes were identified as CD45^hi^/CD115^hi^/Ly6-C/G^lo^ and subdivided into Ly6-C^hi^ (pro-inflammatory) and Ly6-C^lo^ (anti-inflammatory). Neutrophils were identified as CD45^hi^/CD115^lo^/Ly6-C/G^hi^.

#### Heart

Phenotypic macrophage and neutrophil cell and gene expression analyses were conducted in the heart as previously described^22^. Briefly, heart tissue was minced then digested using Collagenase B (Roche, Indianapolis, IN) and Dispase II (Roche, Indianapolis, IN) mixture at 37 °C for 30 min, with gentle agitation every 5–10 min. Digested tissue was filtered using a 100 μm cell strainer, followed by centrifugation at 400×*g* for 5 min at 4 °C. Cells for flow cytometry were incubated immediately with conjugated primary antibodies against Ly6G, CD206, F4/80, CD11b, CD11c, CD45, Ly6C/G, and CD115 for 30 min on ice. CD45^hi^/Ly6G^lo^/F4-80^hi^ cells were identified as macrophages and further classified as pro-inflammatory or reparative based on the expression of CD206 and CD11c. Neutrophils were defined as CD45^hi^/CD115^lo^/Ly6-C/G^lo^ and CD206 was used to further identify N1 neutrophils (CD206^lo^)^39^.

All flow cytometry samples were acquired using an LSR II system (Becton Dickinson) in the University of Kentucky Flow Cytometry Core. We utilized FlowJo (version 7) software to generate dot plots and analyze the data (Becton Dickinson).

### Histology

Histological analysis was performed on deparaffinized and rehydrated sections as previously described^22^. Briefly, at 30 days post-MI, mice (N = 6–10/treatment group) were sacrificed and hearts were isolated and perfused with PBS (VWR International) then by 10% buffered formalin (VWR International) at 75 mmHg. Perfused hearts were imbedded in paraffin and stained with Masson’s trichrome to evaluate scar size. Digital images of stained sections were acquired and analyzed using NIH ImageJ (version 7) software. Scar size was presented as a percentage of the total LV volume.

### Immunohistochemistry

Immunostaining of heart sections was performed on deparaffinized and rehydrated sections as previously described^22^. Briefly, after deparaffinization and rehydration, sections were incubated with primary antibodies against IBA1, CD206 and IL-1β (Sigma Aldrich) followed by secondary antibodies conjugated to Alexa Fluor 488 or 594 (Invitrogen). In the peri-infarct area, 10–15 adjacent zones per sections (1–2 sections/animal) were imaged at 40× magnification utilizing Nikon Confocal Microscope A1 in the University of Kentucky Confocal Microscopy facility. Findings are presented as total number of positive cells per high power field in the area of interest. Using a similar protocol, heart sections (N = 5–7/treatment group) were stained with FITC-conjugated isolectin B4 (FL1201, Vector Labs, Burlingame, CA) to evaluate capillary density. Quantification was performed using Cell Counter plugin for Nikon NIS-Elements (version AR 3.2) in 10–15 adjacent peri-infarct sections. Findings are presented as total capillary density per mm^2^ in the peri-infarct zone. Cell apoptosis was examined using TUNEL and caspase-3 staining in the peri-infarct region. Quantification was performed using Cell Counter plugin for ImageJ (version 1.51d). Findings are presented as total positive cells per high power field in the peri-infarct region. All histological and immunohistochemical assessments were performed by a blinded observer.

### Reverse transcription polymerase chain reaction (RT-PCR)

After RBC lysis, aliquots of 1 × 10^6^ cells were incubated with lysis buffer (Life technologies) and used for gene expression analyses. PureLink RNA Mini Kit (ThermoFisher Scientific) was used to isolate total mRNA from heart and blood cell lysates according to manufacturer protocol. Next, cDNA was generated using SuperScript VILO cDNA synthesis kit (Invitrogen). Using a QuantaStudio 7 Flex real-time thermocycler (Applied Biosystems by life technology), Reverse Transcription-Polymerase Chain Reaction (RT-PCR) was performed to measure the mRNA expression of markers identifying: inducible nitric oxide synthase (iNOS), tumor necrosis factor alpha (TNF-α), monocyte chemotactic protein-1 (MCP-1), transforming growth factor beta (TGF-β), interleukin-1 beta (IL-1β), interleukin-6 (IL-6), interleukin-4 (IL-4), chitinase-like3 Chil3 (YM1), interleukin-10 (IL-10), and Peroxisome proliferator-activated receptor gamma (PPARγ). We used the comparative Ct method for relative estimation of mRNA expression, which was normalized to 18s (a housekeeping gene). Strategies to avoid bias and error inducible by contaminated DNA were taken.

### Echocardiography

Given the fact that the left ventricular appearance in histological sections is affected by multiple technical issues such as perfusion pressure and imbedding techniques in paraffin, we opted to use echocardiography for all left ventricular internal diameter and volume measurements. Mice were anaesthetized using 1–2% isoflurane during echocardiography to maintain heart rate of 450–500 BPM during imaging. A Vevo 3100 system coupled with a 15–7-MHz linear broadband transducer was used to perform Echocardiogram analyses. Heart function was examined at baseline (before cardiac surgery) then at 48 h and 4 weeks post-MI. Using modified parasternal long-axis and short-axis, two-dimensional echocardiography was used to assess the LV function and volume in M-mode. Echocardiography imaging and analyses were carried out by a blinded investigator.

### Luminex assay

At 1- and 3-days post-MI, inflammatory biomarkers (IL-1β, IL-1α, TNF-α, MIP-1α, MIP1β, MIP2, IP-10, and KC) were quantified in plasma using the Milliplex mouse cytokine magnetic kit (MILLIPLEX MAP for Luminex xMap Technology, Millipore, USA) according to the manufacturer’s protocol.

### In vivo fluorescence imaging

Mice were imaged using the Maestro imaging system and an orange filter (excitation 605 nm, emission 675 nm long pass) to capture liposomal APC signal. Images were analyzed using Nikon NIS-Elements AR (New York, USA).

### Cell culture

We used a murine macrophage cell line J774 (ATCC, Manassas, VA), for in vitro experiments examining the immunomodulatory effects of free and liposomal AZM. Cells were plated in 6 well plates at a concentration of 0.3 × 10^6^ cells/well and cultured in DMEM media (supplemented with 10% FBS, 1% penicillin/streptomycin, 1% sodium pyruvate, L-Glutamine, and Glucose). After adhesion, cells were treated with 30 μM free or liposomal AZM (Sigma-Aldrich, St. Louis, MO) and 20 ng/ml IFNγ (eBioscience 14-8311-63). Following overnight incubation at 37 °C with 5% CO_2_, cells were stimulated using 100 ng/ml of LPS (Invivogen). Supernatants were collected 48 h after stimulation to quantify pro- and anti-inflammatory cytokine (TNF-α and IL-10) concentrations.

### ELISA assays

Concentrations of TNF-α and IL-10 were assessed in the supernatant from in vitro cell culture experiments using standard ELISA kits (BD Biosciences, San Deigo, CA) according to the manufacturer protocol. Results are presented for each cytokine (picogram/ml) following different treatments.

### Non-invasive electrocardiogram (ECG) system

Mice were anesthetized with 2% isoflurane, then placed on the ECG platform. Electrodes were inserted subcutaneously on the two front paws and the left rear paw, and the ECG was recorded for 2–3 min. Once the recording was completed, the data were analyzed using Chart software (PhysioTel).

### Statistical analysis

All animals were analyzed separately, and the data is presented as means ± standard error of means (SEM). Unpaired Student t test or analysis of variance (one-way or repeated measures) were used for group comparisons, as appropriate. Two-sided Dunnett or Dunn tests for post hoc multiple comparison procedures were used, with control samples as the control category. P value less than 0.05 was considered statistically significant during the analyses. Statistical analyses were performed using the Prism 8 software package (GraphPad, La Jolla, CA).

## Results

### L-AZM formulation accumulates in the injured myocardium, particularly in immune cells

To address the limited bioavailability and systemic side effects of immunomodulatory drugs after MI, we elected to use non-PEGylated liposomes which are readily engulfed by phagocytes at sites of tissue injury^38^. First, we were interested in determining whether L-AZM accumulates in the injured heart after systemic administration. We used the Maestro EX in vivo imaging system in combination with APC-labeled liposomes for their visualization. We observed significant accumulation of L-AZM in the injured heart as early as the first day after infarction peaking at day 3 (Fig. 1B). This peak coincided with the accumulation of phagocytes in the heart as we and others have shown^9^. Importantly, no signal was observed in the heart of vehicle treated or naive mice either in vivo or ex vivo (Fig. 1B).

The post-infarcted myocardium contains diverse cell populations that may be targeted therapeutically to enhance cardiac recovery^26^. Flow cytometry analysis of APC-labeled L-AZM, delivered intravenously (IV), showed liposomal accumulation in phagocytes such as neutrophils and macrophages beginning on day 1 post-MI. Importantly, less than 1% of non-immune cells in the heart showed liposomal accumulation, implying preferential liposomal accumulation in the cells of interest (Fig. 1C). Compared to IV delivery, we observed delayed liposomal accumulation with intraperitoneal (IP) administration when tested on a subset of animals 24 h after AMI (3 animals/group); hence, we conducted all in vivo studies using IV L-AZM. These findings strongly indicated that our designed non-PEGylated liposomes accumulate at the peri-infarct region, particularly in phagocytic cells, early after MI regardless of approach. Additionally, liposomal accumulation exhibited temporal trends consistent with infiltration of pro-inflammatory cells.

### Liposomal formulation reduces the risk of AZM-induced cardiotoxicity

Clinical studies have suggested small but measurable risk of ventricular arrhythmias following AZM administration in humans^23^. Therefore, we tested the effect of free and liposomal AZM on murine hearts after MI in a subgroup of mice on day 1 post-MI. Baseline parameters were similar among the treatment groups but the IV administration of F-AZM at 40 mg/kg markedly reduced heart rate, RR interval, ejection fraction, and cardiac output compared with baseline (Fig. 1D). Moreover, the QT interval was prolonged after F-AZM administration. These changes were associated with greater than 50% mortality among mice treated with F-AZM (Fig. 1E). In contrast, administration of L-AZM at the same dose did not induce any noticeable conduction, electrical or functional changes in vivo (Fig. 1D). Given the increased mortality with high dose F-AZM, this group was not included in long-term functional recovery studies. Additionally, we examined the potential renal and liver toxicity of liposomal formulations after MI. our analyses did not show significant changes in body weight, renal or liver function assays (Suppl Fig. 1).

We then examined the survival rate after MI in mice treated with L-AZM or vehicle. Survival rates indicated that the cardioprotective effects of L-AZM were translated into a remarkable reduction in mortality (Fig. 1E). These findings imply that L-AZM is indeed protective against the free drug’s adverse hemodynamic effects, which could be, at least in part, attributed to the low liposomal accumulation in cardiomyocytes. Taken together, liposomal encapsulation of AZM is an attractive and safe delivery tool for continued investigation in clinical practice, especially in high risk patients after MI.

### L-AZM therapy shifted macrophage phenotype in the infarcted myocardium

**Figure 2:**
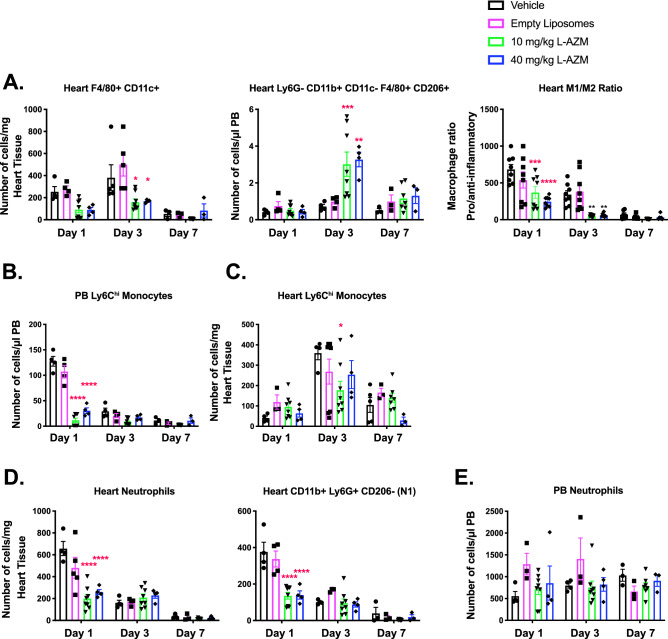

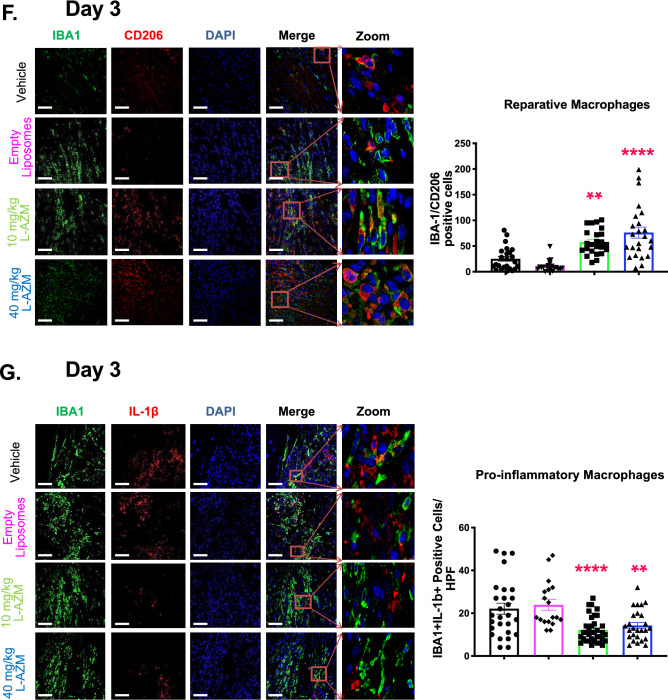
L-AZM treatment reduces inflammatory cells and shifts macrophages to the reparatory phenotype. (**A**) Quantitative FACS data suggests that pro-inflammatory macrophages (F4/80+/CD11c+) markedly decrease in L-AZM treated groups relative to controls starting at day 1, with more profound effect on day 3 post-MI. In contrast, the reparatory macrophages (CD206+) increased significantly at day 3 post-MI in the same treatment groups. These immunomodulatory effects translate into a significant reduction in the pro-/anti-inflammatory macrophage ratio. (**B**) Flow cytometry analysis of peripheral blood pro-inflammatory monocytes (Ly6Chi) demonstrating significant reduction in the L-AZM treated group 3 days post-MI. (**C**) Quantitative analysis of cardiac pro-inflammatory monocytes showing their significant reduction in L-AZM treated mice. (**D**) FACS analyses suggest that neutrophil counts are significantly decreased in the heart, specifically, N1 (CD11b+Ly6G+CD206−) neutrophils, in L-AZM treated mice. (**E**) No significant change was observed in circulating pro-inflammatory neutrophils in our flow cytometry analyses (**F**) Representative images from controls and L-AZM treated mice illustrating IBA1 positive (green) and CD206 positive (red) macrophages in peri-infarct zone. Images show increased expression of CD206 by macrophages in AZM treated groups compared to controls. Quantitative assessment of IBA1 and CD206 3-days post-MI. (**G**) Representative images from controls and L-AZM treated mice illustrating IBA-1 (green) and IL-1β (red) positive macrophages in peri-infarct zones. Images indicate a marked reduction in the expression of IL-1β in macrophages with L-AZM treatment compared to controls. Quantitative assessment of IBA-1 and IL-1β 3-days post-MI (n = 4 animals/group, ***P < 0.001 and ****P < 0.0001 compared to vehicle control). Data presented as mean ± SEM. L-AZM, liposomal azithromycin; PB, peripheral blood. Scale bars represent 50 pm.

We evaluated the effect of L-AZM on the phenotype and function of cardiac macrophages, the primary cardiac immune cells during steady state and after ischemic injury^40^. Our studies focused on a clinically relevant dose of L-AZM (40 mg/kg/day). We also examined the ability of liposomal formulation to enhance the efficacy of a lower L-AZM dose (10 mg/kg/day). We then used flow cytometry to examine the dynamic changes in macrophage phenotypes in the injured heart. We observed a decrease in the number of pro-inflammatory macrophages (CD45^+^/Ly6G^−^/F4-80^+^/CD11c^+^) at days 1 and 3 post-MI in L-AZM treated groups compared to control groups. Conversely, reparative macrophages (CD45^+^/Ly6G^−^/F4-80^+^/CD11c^−^/CD206^+^) were significantly increased at day 3 in L-AZM treated groups compared to controls (Fig. 2). Collectively, these changes translate into a significant reduction in the ratio of pro-/anti-inflammatory macrophages in both L-AZM groups, indicating a shift in macrophage phenotype towards the reparative state (Fig. 2A). Monocytes dominate the infarcted heart following MI, providing a rich source of macrophages^41^. We observed a significant reduction in pro-inflammatory Ly6C^hi^ (CD45^+^/Ly6C/G^hi^/CD115^hi^) monocytes in the PB and heart specifically in the early time points after MI in L-AZM treated groups (Fig. 2B,C). Interestingly, low and high dose of L-AZM offered similar immunomodulatory effects suggesting a broad effective dosing range with liposomal encapsulation.

We then sought to examine the spatial distribution of polarized macrophages in relation to the peri-infarct region. Using immunohistochemistry (IHC), we quantified the number of cells expressing a general macrophage marker (IBA1), a reparative macrophage marker (CD206^+^), and IL-1β as a marker of pro-inflammatory macrophages on day 3 post-MI. We found a significant increase in reparative macrophages (IBA1^+^/CD206^+^) in L-AZM treated mice compared to control groups (Fig. 2F). Conversely, we observed significantly higher numbers of pro-inflammatory macrophages (IBA1^+^/IL-1β^+^) in the control groups (Fig. 2G). Generally, the total number of macrophages (IBA1^+^ cells) were not different between groups, implying that L-AZM therapy does not affect total macrophage count, but rather influences their polarization in favor of a reparative phenotype. These effects may be therapeutically harnessed to resolve the detrimental inflammation post-MI.

### L-AZM treatment reduces cardiac neutrophil influx post-MI

Neutrophils orchestrate the clearance of dead cells and debris^42^. Additionally, apoptotic neutrophils initiate alternative macrophage polarization thus marking the resolution of the post-MI pro-inflammatory phase^8^. Neutrophil (CD45^+^/CD115^lo^/Ly6G/C^lo^) enumeration using flow cytometry revealed that L-AZM treatment significantly reduced cardiac neutrophil count during their peak accumulation at day 1 (Fig. 2D). To further investigate the immunomodulatory actions of L-AZM, we examined the distribution of the pro-inflammatory N1 neutrophils (CD11b^+^/Ly6G^+^/CD206^−^)^39^ in the heart. We observed a substantial decrease in the number of N1 neutrophils at day 1 in the L-AZM treated groups (Fig. 2D), which may explain the reduction in total neutrophil number. We did not observe noticeable differences in PB neutrophils among treatment groups (Fig. 2E). These findings point to a novel mechanistic pathway of the immunomodulatory effects of L-AZM.

### L-AZM treatment shifts inflammatory gene expression towards the anti-inflammatory state

**Figure 3:**
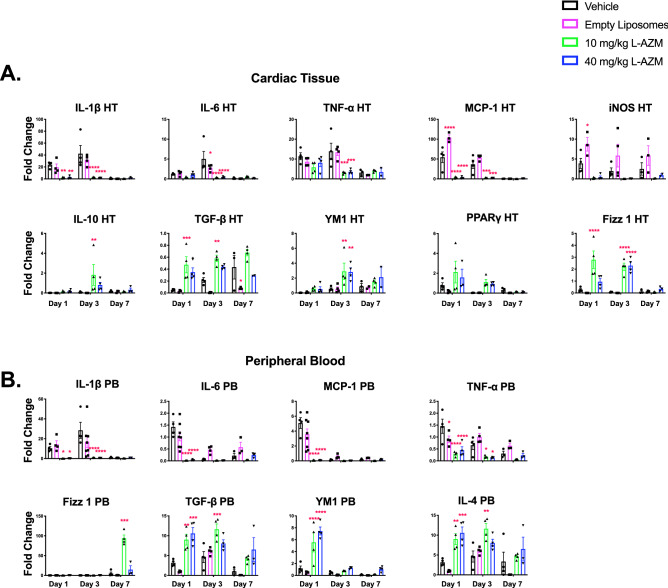
L-AZM modulates pro- and anti-inflammatory cytokine expression in the heart. Heart homogenate (HT) cells were used to quantify pro-inflammatory cytokine expression via RT-PCR. (**A**) Pro-inflammatory cytokines: iNOS, TNF-α, MCP-1, IL-1 β,and IL-6 show significant reduction in expression levels at days 1 and 3 with L-AZM treatment in HT. Anti-inflammatory cytokines: IL-10, TGF-β, Fizz1, YM1, and PPARγ expression were significantly increased on days 1, 3 and 7 with L-AZM treatment. (**B**) Similar to changes observed in cardiac tissue, peripheral blood cells show significant reduction in pro-inflammatory cytokine expression, including TNF-α, MCP-1, IL-1 βand IL-6 at days 1 and 3, and 7 with L-AZM treatment. On the other hand, the expression of anti-inflammatory cytokines on days 1, 3 and 7 were significantly increased with L-AZM treatment in peripheral blood (n = 4 animals/group/time point, *P < 0.05, **P < 0.01, ***P < 0.001, and ****P < 0.0001 compared to the vehicle control). Fold change relative to sham control. Data presented as mean ± SEM. L-AZM, liposomal azithromycin; HT, heart; IL-1β, interleukin 1 beta; IL-6, interleukin 6; iNOS, inducible nitric oxide synthase; Fizz1, found in inflammatory zone 1; MCP-1, monocyte chemoattractant protein-1; PB, peripheral blood; PPARγ, peroxisome proliferator-activated receptor gamma; TGF-1β, tissue growth factor 1 beta; TNF-α, tumor necrosis factor-alpha, YM1 (Chil3), chitinase-like 3; IL-10, interleukin-10.

Pro-inflammatory macrophages produce large quantities of inflammatory cytokines (IL-1β, TNF-α and IL-6) and toxic effector molecules (reactive oxygen species and nitric oxide)^43^, contributing to infarct expansion. Likewise, reparative macrophages are potent generators of anti-inflammatory cytokines (TGF-β and IL-10) that contribute to scar maturation and tissue healing^44^. We assessed the mRNA expression of pro-inflammatory and reparatory genes in the heart and PB cells using RT-PCR. We observed a significant shift in the gene expression profile towards the anti-inflammatory state associated with L-AZM treatment. In cardiac tissue, mRNA levels of iNOS (pro-inflammatory macrophage marker) and pro-inflammatory cytokines (MCP-1, TNF-α, IL-6, and IL-1β) were substantially downregulated in mice treated with L-AZM, particularly on days 1 and 3 post-infarct (Fig. 3A). Conversely, the expression of anti-inflammatory cytokines and reparative genes (TGF-β, IL-10, Fizz1, PPARγ, and YM1) were significantly upregulated in the same groups of mice with consistent effects up to day 7. Interestingly, we observed a similar trend in PB cells in L-AZM treated groups (Fig. 3B), suggesting systemic anti-inflammatory and immunomodulatory effects of L-AZM therapy. As seen with the number of inflammatory cells, both low and high L-AZM doses demonstrated equivalent immunomodulatory effects.

We conducted additional studies examining the protein levels of inflammatory cytokines in PB at the same time points after MI. We observed that the PB inflammatory cytokines and chemokines (IL-1β, IL-1α, TNF-α, MIP-1α, MIP1β, MIP2, IP-10, and KC) were reduced in plasma of L-AZM treated groups during the peak inflammatory period (Suppl Fig. 1). To identify whether the liposomal formulation enhanced the anti-inflammatory effects of AZM, we conducted in vitro experiments to identify the production of pro- and anti-inflammatory cytokines in the J774 macrophage cell line. After 48 h of LPS stimulation, we observed accentuated immunomodulatory effects with L-AZM, confirmed through the significant reduction in TNF-α and increase in IL-10 production (Suppl Fig. 2). Collectively, these results from multiple in vitro and in vivo studies suggest that L-AZM has strong immunomodulatory effects and that liposomal encapsulation conveys therapeutic clinical potential in MI.

### L-AZM therapy reduces cell death and scar size and increases angiogenesis in the infarcted heart

**Figure 4:**
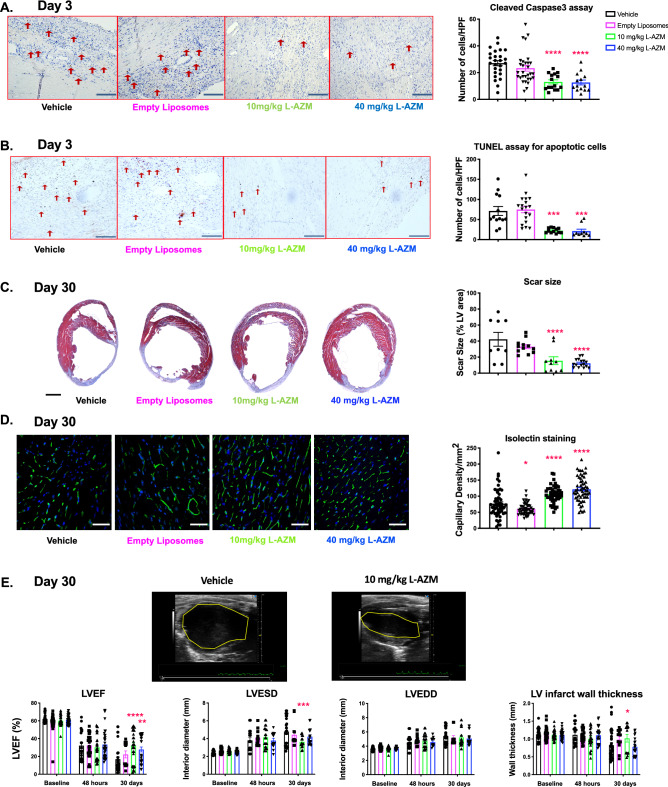
Liposomal AZM reduces cell death and enhances cardiac recovery post-infarction. (**A**) Representative light microscope images of caspase-3 staining for peri-infarct regions in controls and L-AZM treated mice 3 days post-MI. Quantitative analyses of caspase-3 positive cells in L-AZM treated groups compared to the vehicle control group (n = 4 animals/group, scale bars represent 100 μm). (**B**) Representative light microscope images of TUNEL staining of peri-infarct regions in controls, free AZM, and L-AZM treated mice 3 days post-MI. Quantitative analysis of apoptotic cell numbers among treatment groups (n = 4 animals/group, scale bars represent 100 μm). (**C**) Representative Masson's trichrome staining at 30 days post-MI in control and L-AZM treated groups and quantitative analysis of scar as a percentage of LV area for each group (n = 6–10 animals/ group, scale bars represent 200 μm). (**D**) Representative isolectin staining (Green), as surrogate for capillary density, at 30 days post-MI in the peri-infarct region in control, F-AZM, and L-AZM treated animals. Quantitative analysis of capillary density confirms a higher rate of angiogenesis and capillary density in L-AZM treated groups (n = 5–10 animals/group, scale bars represent 50 μm). (**E**) Representative images demonstrating the left ventricular cavity size between groups (endocardium border is marked using the yellow dashed line). Quantitative analyses demonstrate significant recovery in LV function as assessed by ejection fraction (LVEF), which significantly improved in L-AZM groups. Data also shows significant improvement in LV adverse remodeling parameters such as end-systolic diameter (LVESD) and end-diastolic diameter (LVEDD). (n = 6–10 animals/ group). Data presented as mean ± SEM. F-AZM, free formulation of azithromycin, L-AZM, liposomal azithromycin. *P < 0.05, ***P < 0.01, ***P < 0.001 and ****P < 0.0001 compared to the vehicle control.

To identify the pro-survival effect of L-AZM therapies on cardiac cells post-MI, we assessed apoptosis in the peri-infarct region 3 days post-MI using caspase-3 (early apoptosis) and TUNEL (late apoptosis) staining. We observed that L-AZM therapy markedly reduced early and late apoptosis in comparison to vehicle and empty liposome controls (Fig. 4A,B), likely related to pro-survival factors produced by reparative macrophages^45^. We next assessed the infarct size 30 days post-MI using Masson’s trichrome-stained sections from the heart. We found that both doses of L-AZM treatment significantly reduced scar size in comparison to controls (Fig. 4C). These data indicate that L-AZM preserves the myocardium from acute ischemic injury as well as reduces susceptibility to chronic scar expansion.

Reparative macrophages promote cardiac recovery through the secretion of protective cytokines and pro-angiogenic factors such as VEGF^45^. To determine blood vessel density at the infarct borders, we quantified isolectin-positive cells (a marker of endothelial cells) in the peri-infarct region 30 days post-MI. We observed a significant increase in capillary density in the L-AZM groups compared to controls (Fig. 4D). Together with reduced cell apoptosis, enhanced angiogenesis can explain the reduction in scar size observed with L-AZM therapy.

### L-AZM therapy enhances cardiac functional recovery after MI

Adverse cardiac remodeling and the ensuing heart failure post-MI are likely related to an imbalance between pro-inflammatory and reparative phases resulting in ineffective tissue healing^18^. We performed echocardiography at 1- and 4-weeks post-MI and analyzed cardiac function and remodeling. We observed a significant attenuation of LV functional decline in L-AZM treated groups compared to controls (Fig. 4E). A similar trend was noted with cardiac remodeling parameters such as LV end-systolic and LV end-diastolic diameters in the same groups (Fig. 4E), which is consistent with our histological examination. Collectively, the immunomodulatory effects of L-AZM therapy were associated with reduction in cell death and enhanced angiogenesis. These salutary effects led to effective tissue healing and improved cardiac functional recovery.

## Discussion

Modulating inflammation after MI via alternative macrophage polarization, is associated with improved cardiac functional recovery and survival. However, there are no clinically approved therapies targeting this critical pathway to date. We recently demonstrated that AZM improves cardiac remodeling and recovery post-MI through the shifting of macrophages to the reparatory state in a proof-of-concept experimental model^22^. In the current study, we utilized a clinically relevant experimental design using liposomal delivery to potentiate AZM bioavailability and immunomodulatory effects post-MI. We also harnessed the phagocytic capacity of monocytes and macrophages to rapidly recognize the non-PEGylated liposomal formulation and improve immune cell selectivity and polarization. L-AZM promoted resolution of post-MI inflammation as evidenced by a shift from a pro-inflammatory monocyte/macrophage dominance to a response consisting predominantly of reparative macrophages. These cellular changes were observed primarily at the site of cardiac injury and were paralleled by a shift towards anti-inflammatory cytokine production and cellular preservation; leading to reduced scar size, enhanced angiogenesis, and improved cardiac functional recovery. This study is the first to describe the immune modulatory properties of L-AZM and to demonstrate that liposomal encapsulation is significantly protective from adverse cardiac effects. Our findings imply that low dose, post-MI treatment with L-AZM is a promising therapeutic intervention in patients with MI.

Several therapeutic agents have been tested to alleviate cardiac remodeling post-MI through systemic administration; however, they produced limited efficacy due to poor accumulation in the target site/cells, incompatible pharmacokinetic properties with MI, or dose-limiting adverse effects^26^. Therefore, an optimal drug delivery system is needed to achieve higher therapeutic concentration in the injured myocardium while maintaining low off-target effects^26^. Using in vivo imaging and flow cytometric analyses, we confirmed preferential liposomal accumulation in phagocytes post-MI, particularly in the first 24 h. Importantly, our data suggest that liposomes accumulate in less than 1% of non-immune cells. This distribution likely drives our observed increase in on-target effects (enhanced efficacy) and reduction in off-target effects (less potential for cardiac adverse effects). Indeed, our in vivo electrophysiological monitoring confirmed cardiac protection with L-AZM against conduction abnormalities. However, more studies may be needed to further refine drug delivery and release in the setting of MI.

We previously observed the immunomodulatory effectiveness of AZM as a potential therapy for cardioprotection after ischemic injury^22^. In this earlier proof-of-concept study, pre-MI treatment and a relatively high dose of AZM (160 mg/kg/day) were used to provide time for the drug to reach steady levels before MI^46,47^. To avoid these translational barriers and to improve the drug potency^38,48^, we considered an encapsulated formulation of AZM. Our data demonstrate enhanced pharmacological effects of liposomal formulations with both low (10 mg/kg) and high dose (40 mg/kg) of L-AZM. While our data suggest enhanced immunomodulatory effects achieved by the liposomal formulation, we could not directly confirm the concentration of AZM in immune cells. Therefore, we are currently working on a biotinylated form of AZM that would allow us to track and quantify this drug both in vivo and in vitro using different encapsulation strategies.

It is widely accepted that neutrophils are the first immune cells to infiltrate the infarcted heart and their extended presence is associated with exacerbation of cardiac injury after ischemia^49^. While neutrophils play an important role in removing dead cells and debris, their byproducts may exacerbate cardiac damage. Neutrophils can also negatively impact post-MI healing through the preferential recruitment of pro-inflammatory (Ly6C^hi^) monocytes^50^. A recent study reveals two activation states of neutrophils in the post-ischemic heart, namely an early pro-inflammatory and potentially deleterious (N1) and a late anti-inflammatory (N2) pro-reparatory population of neutrophils^39^. Here, we observed that the number of N1 neutrophils is significantly decreased with L-AZM therapy, which could be attributed to the apoptotic effects of the drug or reduced neutrophil recruitment due to suppressed production of pro-inflammatory chemokines as we observed here and in our previous work^22^. Additionally, we observed a significant reduction in cardiac Ly6C^hi^ monocytes with L-AZM therapy, which is most likely related to the diminished expression of MCP-1 in the heart and its reduced levels in the PB.

The post-MI healing phase is primarily organized by reparative macrophages^11^. This could explain the attenuated cardiac recovery observed with macrophage depletion and the improved outcomes with the adoptive transfer of alternatively activated macrophages^51,52^. Furthermore, multiple studies have shown the therapeutic utility of shifting macrophages to the reparative phenotype post-MI^53,54^. AZM facilitates the early transition to anti-inflammatory macrophages and inflammation resolution through its effects on NF-kb and STAT1 signaling^55^. We observed that AZM therapy accelerates neutrophil apoptosis, which could explain the shift in macrophage polarization state to the anti-inflammatory phenotype^22^; as macrophages phagocytose apoptotic cells, they initiate the internal machinery to switch their phenotype to anti-inflammatory cells^56^. We found that L-AZM treatment was associated with higher anti-inflammatory (IBA1^+^/CD206^+^) and lower pro-inflammatory (IBA1^+^I/L-1β^+^) macrophages after MI, particularly in peri-infarct regions. Additionally, we observed a significant reduction in apoptosis at day 3 post-MI in the same regions attributed to pro-survival factors released from reparative macrophages^57,58^. On the other hand, anti-inflammatory genes such as TGF-β, IL-4, and IL-10, as well as reparative macrophage markers such as FIZZ1, YM1, PPAR-ɣ, and ARG were upregulated by L-AZM treatment. These mediators exert multiple beneficial functions in the myocardium following MI, orchestrating the transition from the inflammatory to the healing phase^59^. Reduction in post-MI inflammation induced by L-AZM treatment translated into smaller scar, enhanced angiogenesis and better cardiac functional recovery after MI.

## Conclusion

This is the first report utilizing this novel and clinically relevant strategy of liposomal formulation of azithromycin for immunomodulation that could potentially be extended to other systems. It is expected that liposomes would be beneficial as anti-inflammatory drug carriers in other sterile inflammatory diseases that share similar inflammatory profile with MI such as ischemic stroke and spinal cord injury^47,60^. Furthermore, the reduction in cardiac off-target effects highlight the benefit of liposomal formulation of AZM for clinical applications. Our findings strongly support clinical trials using L-AZM as a novel and clinically relevant therapy to improve cardiac recovery and reduce heart failure post-MI in humans.

## Supplementary information

Supplementary Information.
