# Author Correction: Liposomal delivery of azithromycin enhances its immunotherapeutic efficacy and reduces toxicity in myocardial infarction

**DOI:** 10.1038/s41598-021-85720-6

**Published:** 2021-03-17

**Authors:** Ahmed Al‑Darraji, Renée R. Donahue, Himi Tripathi, Hsuan Peng, Bryana M. Levitan, Lakshman Chelvarajan, Dalia Haydar, Erhe Gao, David Henson, John C. Gensel, David J. Feola, Vincent J. Venditto, Ahmed Abdel‑Latif

**Affiliations:** 1 Gill Heart and Vascular Institute and Division of Cardiovascular Medicine, University of Kentucky, Lexington, KY USA; 2 College of Pharmacy, University of Kentucky, Lexington, KY USA; 3 The Center for Translational Medicine, Temple University School of Medicine, Philadelphia, PA USA; 4 Spinal Cord and Brain Injury Research Center, Department of Physiology, College of Medicine, University of Kentucky, Lexington, USA; 5 Division of Cardiology, University of Kentucky and the Lexington VAMC, 741 S. Limestone Street, BBSRB, Room 349, Lexington, KY 40536-0509 USA

Correction to: *Scientific Reports* 10.1038/s41598-020-73593-0, published online 06 October 2020

The Acknowledgements section in this Article is incomplete.

“The UK Flow Cytometry & Cell Sorting core facility is supported in part by the Office of the Vice President for Research, the Markey Cancer Center and an NCI Center Core Support Grant (P30 CA177558) to the University of Kentucky Markey Cancer Center.”

should read:

“The UK Flow Cytometry & Cell Sorting core facility is supported in part by the Office of the Vice President for Research, the Markey Cancer Center and an NCI Center Core Support Grant (P30 CA177558) to the University of Kentucky Markey Cancer Center. V.J.V. was supported by the University of Kentucky Center for Biomedical Research Excellence (COBRE) in Pharmaceutical Research and Innovation (CPRI, NIH P20GM130456) and a grant from the National Institutes of Health (R01HL152081).”

