# Supplementary material for: Liposomal delivery of azithromycin enhances its immunotherapeutic efficacy and reduces toxicity in myocardial infarction
Source: Sci Rep. 2020 Oct 6;10:16596. doi: 10.1038/s41598-020-73593-0 (PMC7538891; doi:10.1038/s41598-020-73593-0)
Supplement: Supplementary file 1 — Supplementary Information. [file 41598_2020_73593_MOESM1_ESM.pdf]

Liposomal Delivery of Azithromycin Enhances its Immunotherapeutic Efficacy and Reduces Toxicity in Myocardial Infarction

Ahmed Al-Darraji[1], Renée R. Donahue[1], Himi Tripathi[1], Hsuan Peng[1], Bryana M. Levitan[1], Lakshman Chelvarajan[1], Dalia Haydar[2], Erhe Gao[3], David Henson[2], John C. Gensel[4], David J. Feola[2], Vincent J. Venditto[2], and Ahmed Abdel-Latif[1]

[1]Gill Heart and Vascular Institute and Division of Cardiovascular Medicine, University of Kentucky and the Lexington VA Medical Center, Lexington, KY, USA

[2]College of Pharmacy, University of Kentucky, Lexington, KY, USA

[3]The Center for Translational Medicine, Temple University School of Medicine, Philadelphia, PA, USA

[4]Spinal Cord and Brain Injury Research Center, Department of Physiology, College of Medicine University of Kentucky

Supplemental Data

Materials and method

Study Design

C57BL/6 male mice (Jackson Laboratory, BarHarbor, ME), age 6-8 weeks, were treated with free/liposomal AZM (10 or 40 mg/kg/day) or vehicle (PBS or empty liposomes) using retro-orbital (intravenous-IV) or intraperitoneal (IP) injection, starting immediately after MI or sham surgery and continued for 7 days (**Fig 1A**). This administration strategy was chosen to mimic commonly prescribed AZM regimens in patients being treated for infection[1,2] and provides effective distribution and tissue accumulation[3]. Treatment was continued for 7 days to encompass the duration of the post-MI inflammation[4]. All surviving animals were followed for up to 35 days for survival, cardiac functional recovery and scar formation. All procedures were conducted under the approval of the University of Kentucky IACUC in accordance with the NIH Guide for the Care and Use of Laboratory Animals (DHHS publication No. [NIH] 85–23, rev. 1996).

Murine model of myocardial infarction

Anesthesia was induced through a small animal vaporizer system with 1–3% isoflurane. Pain reflexes were appropriately tested to confirm adequate anesthesia before and during the surgical procedure. Lateral thoracotomy was performed between the left fourth and fifth ribs, the pericardial space was accessed, and the heart protruded from the intercostal space. The left anterior descending coronary artery (LAD) was identified and permanently ligated with 6–0 silk suture 3 mm distal to its origin, as previously described [5]. Sham surgery consisted of the same procedure without LAD ligation. The heart was immediately returned to the intrathoracic cavity and air was manually evacuated to prevent pneumothorax. Finally, the muscle and skin were sutured using a 4–0 Prolene running suture.

Preparation of liposomes

Liposomal formulations were prepared according to a previously established protocol[6,7], using the thin film hydration method with equal molar ratios of distearyl phosphatidylcholine (DSPC), distearyl phosphatidylglycerol (DSPG) and cholesterol (Avanti polar lipids). AZM was included at 10 and 30 mol% based on phospholipid content. Formulations containing fluorophores were prepared in the same manner with 0.5 mol% of the lipophilic dye, 1,1'-dioctadecyl-3,3,3',3'-tetramethylindodicarbocyanine (DiD). Lipids and AZM were mixed in chloroform and methanol at the desired ratio and the organic solvents removed by rotary evaporation to yield a thin film. After being placed under a vacuum overnight, the lipid/drug films were hydrated in phosphate buffered saline (pH 7.4) and sonicated at 65°C for 30 min. The resulting unilamellar vesicles were extruded through 200 nm polycarbonate membranes to obtain uniform particle size. Liposome size, polydispersity and stability over time were determined by dynamic light scatter testing.

Flow cytometry

Peripheral blood (PB). Cell phenotype, in addition to gene analyses, of monocytes and neutrophils was examined in peripheral blood as previously described[5]. Briefly, blood was collected in tubes with a 1:5 ratio of ethylene diaminetetraacetic acid (EDTA)/citrate-theophylline-adenosinedipyridamole (CTAD) on days 1, 3 and 7 post-MI. Whole blood was centrifuged for 5 minutes at 500 x *g*, and the plasma layer was separated and reserved at -80^{0}C. To lyse red blood cells, the residual cell pellet was incubated with 0.5 ml of diluted red blood lysing buffer (BD pharm lyse) for 10 minutes with mild agitation. To end the lysis process, 0.5 ml of staining buffer (5% goat serum, 0.05% sodium azide in phosphate-buffered saline) was added to the suspension and centrifuged at 400 x *g* for 5 minutes, and the supernatant removed. This step was repeated if the red blood cells were still detectable in the cell pellet. Cells were then washed twice in staining buffer to remove any residual lysis buffer. After supernatant removal, the pellet was resuspended in a pre-determined volume of staining buffer and counted. Cells for staining were incubated immediately with conjugated primary antibodies against PERCPCY5.5-conjugated Ly6G/C (BD

Pharmingen), PECY7-conjugated F4/80 (Biolegend), brilliant violet 421-conjugated CD11b (Biolegend), APC-CY7-conjugated CD45 (Biolegend), and PE-conjugated CD115 (Biolegend) for 30 minutes on ice. Following incubation, cells were washed twice with staining buffer and analyzed by an LSR II (Becton Dickinson) in the University of Kentucky Flow Cytometry Core. Using unstained cells and single fluorescent controls, laser calibration and compensations were performed for all experiments. Monocytes with the $CD45^{hi}/CD115^{hi}/Ly6\text{-}C^{hi}$ profile were identified as classical (pro-inflammatory), while cells with these markers $CD45^{hi}/CD115^{hi}/Ly6\text{-}C^{lo}$ were identified as non-classical (anti-inflammatory). Neutrophils were identified as $CD45^{hi}/CD115^{lo}/Ly6\text{-}C/G^{lo}$.

Heart. Phenotypic cell analysis of macrophages and neutrophils and gene expression analyses waere conducted in the heart as previously described[5]. Briefly, mice were sacrificed, and hearts were rapidly isolated and placed in ice cold PBS (VWR International). Using a razor blade, the heart was minced manually. After mincing, tissue was incubated with a Collagenase B (Roche, Indianapolis, IN) and Dispase II (Roche, Indianapolis, IN) mixture at 37°C for 30 minutes, with gentle agitation every 5-10 minutes. Tissue digestion ceased using cold staining buffer and the suspension was placed on ice and filtered using a 100 µm cell strainer, followed by centrifugation at 400 x *g* for 5 mins at 4°C. Supernatant was discarded and the pellet was resuspended in 0.3 ml of staining buffer. Individual heart cells were divided into approximately three equal portions depending on cell counting with $\sim 10^6$ live (trypan blue-negative) cells in each. Cells for flow cytometry were incubated immediately with conjugated primary antibodies against FITC-conjugated Ly6G (BD Pharmingen), PE-conjugated CD206 (Biolegend), PECY7-conjugated F4/80 (Biolegend), brilliant violet 421-conjugated CD11b (Biolegend), brilliant violet 510-conjugated CD11c (Biolegend), APC-CY7-conjugated CD45 (Biolegend), *or* PERCPCY5.5-conjugated Ly6G/C (BD Pharmingen), PECY7-conjugated F4/80 (Biolegend), brilliant violet 421-conjugated CD11b (Biolegend), APC-CY7-conjugated CD45 (Biolegend), and PE-conjugated

CD115 (Biolegend) for 30 minutes on ice. Cells were then washed twice with staining buffer and analyzed by an LSR II (Becton Dickinson) in the University of Kentucky Flow Cytometry Core. Using unstained cells and single fluorescent controls, laser calibration and compensations were performed for all experiments. $CD45^{hi}/Ly6G^{lo}/F4\text{-}80^{hi}$ cells were identified as macrophages and further classified as pro-inflammatory or reparative based on the expression of CD206 and CD11c. Neutrophils were defined as $CD45^{hi}/CD115^{lo}/Ly6\text{-}C/G^{lo}$ and CD206 was used to further identify N1 neutrophils ($CD206^{lo}$).

Histology

Histological analysis was performed on deparaffinized and rehydrated sections as previously described[5]. Briefly, at 30 days post-MI, mice (N = 6-10/treatment group) were sacrificed and hearts were isolated and perfused with PBS (VWR International) then by 10% buffered formalin (VWR International) at 75 mmHg. Perfused hearts were placed in 10% neutral buffered formalin (VWR) overnight at room temperature, then sectioned into 2-mm cross-sections starting at the level of coronary ligation then imbedded in paraffin and sectioned into 4-μm sections. Sections were stained with Masson's trichrome to evaluate scar size as previously described[5]. Digital images of stained sections were acquired, and areas of interest were assessed using NIH ImageJ (version 7) software. The LV area, LV cavity area, and infarct area were measured as previously described[5]. Scar size was presented as a percentage of the total LV volume.

Immunohistochemistry

Immunostaining of heart sections was performed on deparaffinized and rehydrated sections as previously described[5]. Briefly, after deparaffinization, rehydration, and antigen retrieval, sections were incubated with primary antibodies: rabbit anti-mouse IBA1 (Wako), goat anti-mouse CD206 (R and D Systems), and goat anti-mouse IL-1β (R & D Systems) overnight at 4°C, then washed and incubated with secondary antibodies conjugated to Alexa Fluor 488 or 594 (Invitrogen), followed by incubation with DAPI. In the peri-infarct area, 10-15 adjacent zones per

section (1–2 sections/animal) were imaged at 40x magnification utilizing Nikon Confocal Microscope A1 in the University of Kentucky Confocal Microscopy facility. Nucleated cells that were antibody-positive were quantified using Cell Counter plugin for Nikon NIS-Elements (version AR 3.2). Findings are presented as total number of positive cells per high power field in the area of interest.

By using a similar protocol to the above, heart sections (N=5-7/treatment group) were prepared from mice sacrificed at day 30 post-MI to evaluate capillary density. Sections were stained with FITC-conjugated isolectin B4 (FL1201, Vector Labs, Burlingame, CA). In the peri-infarct region, 10-15 adjacent zones per section (1–2 sections/animal) were imaged at 40x magnification utilizing Nikon Confocal Microscope A1. Quantification was performed using Cell Counter plugin for Nikon NIS-Elements (version AR 3.2). Findings are presented as total capillary density per mm^2 in the peri-infarct zone. All measurements were performed in the peri-infarcted areas only and analyzed by blinded observer.

Cell apoptosis was examined on deparaffinized and rehydrated sections from mice (N = 4/treatment group) sacrificed at day 3, as previously described[5]. TUNEL and caspase-3 staining was performed in the Biospecimen Procurement and Translational Pathology Shared Resource Facility (BPTP SRF) at the University of Kentucky. Nucleated cells that stained positive were estimated. Quantification was performed using Cell Counter plugin for ImageJ (version 1.51d). Findings are presented as total number of positive cells per high power field in the peri-infract region. All measurements were obtained in the peri-infarcted areas only and analyzed by blinded observer.

Reverse Transcription Polymerase Chain Reaction (RT-PCR).

After RBC lysis (see above), aliquots of $1x10^6$ cells were incubated with lysis buffer (Life technologies) for 10 minutes with multiple aggressive agitations, and then kept at -80^0C for further gene expression analysis. PureLink RNA Mini Kit (ThermoFisher Scientific) was used to isolate

total mRNA from heart and blood cell lysates according to manufacturer protocol. Isolated RNA was quantified using NanoDrop 8000 spectrophotometer (Thermofisher). Next, cDNA was generated using SuperScript VILO cDNA synthesis kit (Invitrogen). Using a QuantaStudio 7 Flex real-time thermocycler (Applied Biosystems by life technology), Reverse Transcription-Polymerase Chain Reaction (RT-PCR) was performed to measure the mRNA expression of markers identifying: inducible nitric oxide synthase (iNOS), tumor necrosis factor alpha (TNF-α), monocyte chemotactic protein-1 (MCP-1), transforming growth factor beta (TGF-β), interleukin-1 beta (IL-1β), interleukin-6 (IL-6), interleukin-4 (IL-4), chitinase-like3 Chil3 (YM1), interleukin-10 (IL-10), and Peroxisome proliferator-activated receptor gamma (PPARγ). We used the comparative Ct method for relative estimation of mRNA expression which was normalized to 18s (a housekeeping gene). Strategies to avoid bias and error inducible by contaminated DNA were taken: (a) Primers were adjusted to bridge an intron for specific cDNA augmentation, (b) Appropriate negative control reactions (template free controls) were used, (c) Careful examination of the melting curve of augmented products (dissociation graphs) for consistency was performed, and (d) The probe Tm was at least 10°C more than the primer Tm, while the melting temperature (Tm) was 57°C–60°C.

Echocardiography

Given the fact that the left ventricular appearance in histological sections is affected by multiple technical issues such as perfusion pressure and imbedding techniques in paraffin, we opted to use echocardiography for all left ventricular internal diameter and volume measurements. Mice were anaesthetized using 1%–3% isoflurane during echocardiography to maintain heart rate of 450-500 BPM during imaging. A Vevo 3100 system coupled with a 15-7-MHz linear broadband transducer and a 12-5-MHz phased array transducer was used to perform Echocardiogram analyses. Heart function was examined at baseline (before cardiac surgery) then at 48 hours and 4 weeks post-MI. During acquisition, a heating pad was used to preserve the body temperature

at 37°C, continuously assessed by a rectal temperature probe. Using modified parasternal long-axis and short-axis, two-dimensional and Doppler echocardiography was used to assess the LV function and volume in M-mode. Internal dimension tracing was performed at the mid-papillary level to calculate the systolic and diastolic parameters and Teichholz formula were used to quantify LV function. Echocardiography imaging and analyses were carried out by a blinded investigator.

Luminex assay

At 1- and 3-days post-MI, plasma was collected using the PB collection protocol detailed earlier. Inflammatory biomarkers (IL-1β, IL-1α, TNF-α, MIP-1α, MIP1β, MIP2, IP-10, and KC) were quantified using the Milliplex mouse cytokine magnetic kit (MILLIPLEX MAP for Luminex xMap Technology, Millipore, USA) according to the manufacturer's protocol.

I*n vivo* fluorescence imaging

Mice were placed inside the Maestro imaging system under isoflurane anesthesia. They were positioned so that the chest and abdomen of the animal were in the field of view. We used an orange filter (excitation 605nm, emission 675nm long pass) to capture the APC signal that comes from liposomes. Acquisition settings were in range from 640 to 820nm in 10-nm steps. To enhance the quality of the images we adjusted variables including stage height, focus, and exposure length within time points. Images were taken with side-by-side LAZM and vehicle-injected control animals and were analyzed using Maestro software (Cambridge Research & Instrumentation, INC. (CRI), USA).

Cell culture

We used a murine macrophage cell line, J774 (ATCC, Manassas, VA), for *in vitro* experiments examining the immunomodulatory effects of free and liposomal AZM. Using DMEM media (supplemented with 10% FBS, 1% penicillin/streptomycin, 1% sodium pyruvate, L-

Glutamine, and Glucose), cells were plated in 6 well plates at a concentration of 0.3×10^6 cells/well. After adhesion, cells were treated with 30 μM free or liposomal AZM (Sigma-Aldrich, St. Louis, MO) and 20 ng/ml IFNγ (eBioscience 14-8311-63). Following overnight incubation at 37°C with 5% CO2, cells were stimulated using 100 ng/ml of LPS (Invivogen). Supernatants were collected 48 hours after stimulation to quantify pro- and anti-inflammatory cytokine (TNF-α and IL-10) concentrations.

ELISA assays

Concentrations of TNF-α and IL-10 were assessed in the supernatant from in vitro cell culture experiments using standard ELISA kits (BD Biosciences, San Deigo, CA) according to the manufacturer protocol. Results are presented for each cytokine (picogram/ml) with different treatments.

Non-invasive electrocardiogram (ECG) system

Mice were anesthetized with 2% isoflurane, then placed on the ECG platform. Electrodes were inserted subcutaneously on the two front paws and the left rear paw, and the ECG was recorded for 2-3 minutes. Once the recording was completed, the data were analyzed using Chart software (PhysioTel).

Statistical Analysis

Data were presented as mean ± standard error of the mean (SEM). Unpaired Student t test or analysis of variance (one-way or multiple comparisons) were used for group comparisons, as appropriate. Two-sided Dunnett or Dunn tests for post hoc multiple comparison procedures were used, with control samples as the control category. P value less than 0.05 was considered statistically significant during the analyses. Statistical analyses were performed using the Prism 8 software package (GraphPad, La Jolla, CA).

References.

1 Chang, H. T., Evans, C. T., Weaver, F. M., Burns, S. P. & Parada, J. P. Etiology and outcomes of veterans with spinal cord injury and disorders hospitalized with community-acquired pneumonia. *Arch Phys Med Rehabil* **86**, 262-267, doi:10.1016/j.apmr.2004.02.024 (2005).

2 Evans, C. T., Rogers, T. J., Burns, S. P., Lopansri, B. & Weaver, F. M. Knowledge and use of antimicrobial stewardship resources by spinal cord injury providers. *PM R* **3**, 619-623, doi:10.1016/j.pmrj.2011.03.021 (2011).

3 Shepard, R. M. & Falkner, F. C. Pharmacokinetics of azithromycin in rats and dogs. *J Antimicrob Chemother* **25 Suppl A**, 49-60, doi:10.1093/jac/25.suppl_a.49 (1990).

4 Heidt, T. *et al.* Differential contribution of monocytes to heart macrophages in steady-state and after myocardial infarction. *Circ Res* **115**, 284-295, doi:10.1161/CIRCRESAHA.115.303567 (2014).

5 Al-Darraji, A. *et al.* Azithromycin therapy reduces cardiac inflammation and mitigates adverse cardiac remodeling after myocardial infarction: Potential therapeutic targets in ischemic heart disease. *PLoS One* **13**, e0200474, doi:10.1371/journal.pone.0200474 (2018).

6 Kierstead, P. H. *et al.* The effect of polymer backbone chemistry on the induction of the accelerated blood clearance in polymer modified liposomes. *J Control Release* **213**, 1-9, doi:10.1016/j.jconrel.2015.06.023 (2015).

7 Oh, Y. K., Nix, D. E. & Straubinger, R. M. Formulation and efficacy of liposome-encapsulated antibiotics for therapy of intracellular Mycobacterium avium infection. *Antimicrob Agents Chemother* **39**, 2104-2111 (1995).

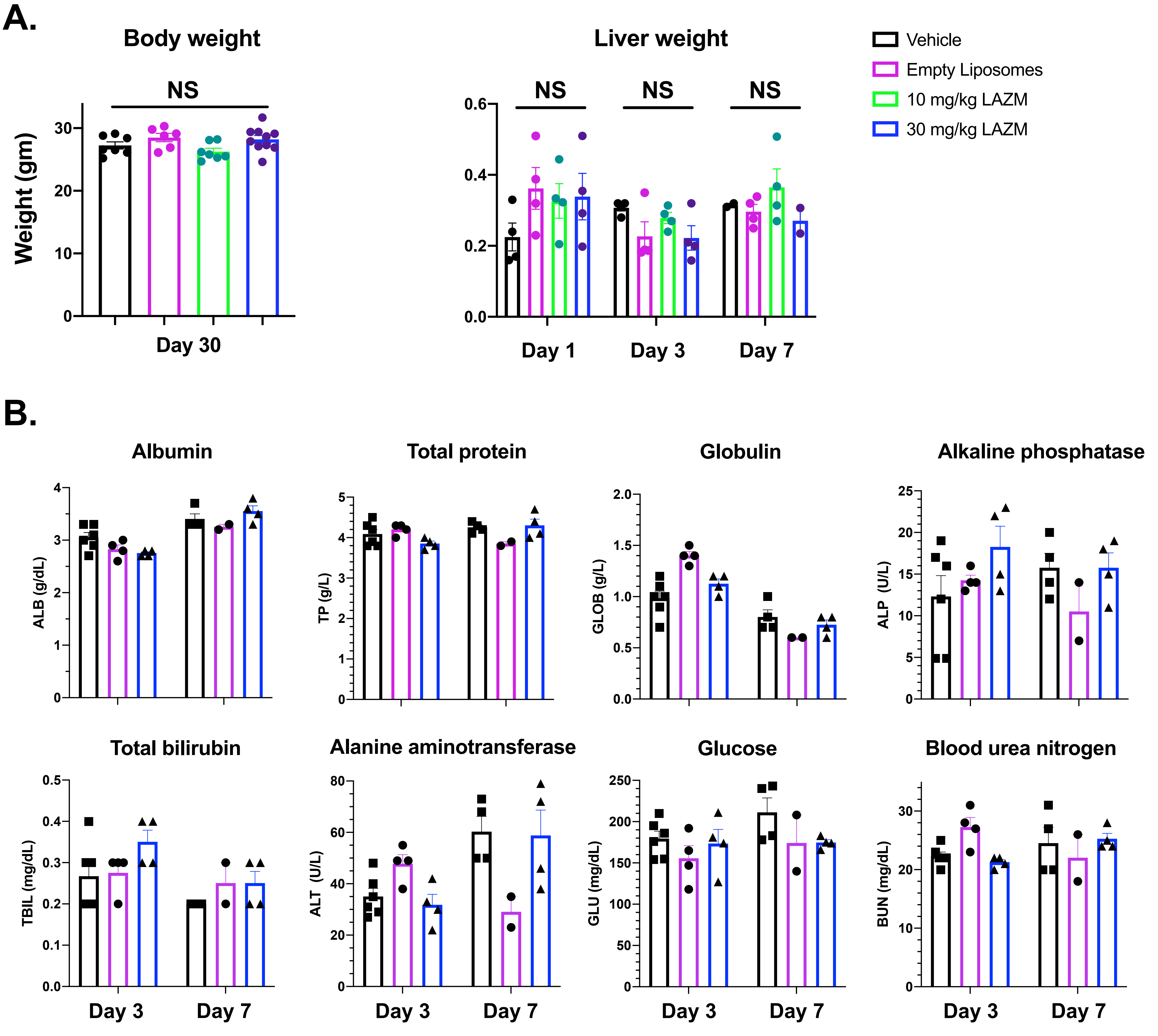

Supplemental Figure 1. Systemic effects of L-AZM. (A) Analysis of body and liver weight among vehicle, empty liposomes and L-AZM formulations (10 and 30 mg/kg) showing no significant differences in body weight at 30 days and liver weight at 1, 3, and 7 days (n = 4-10 animals/group/time point). (B) Results of the complete metabolic panel conducted on plasma isolated from animals from vehicle, low and high LAZM treatment groups at 1, 3 and 7 days after myocardial infarction. The results show comparable plasma protein levels, markers of liver function (alkaline phosphatase, total bilirubin, and alanine transferase), glucose level or kidney function (blood urea nitrogen levels) (N = 2-4 animals/group/time point).

Suppl Fig 2.

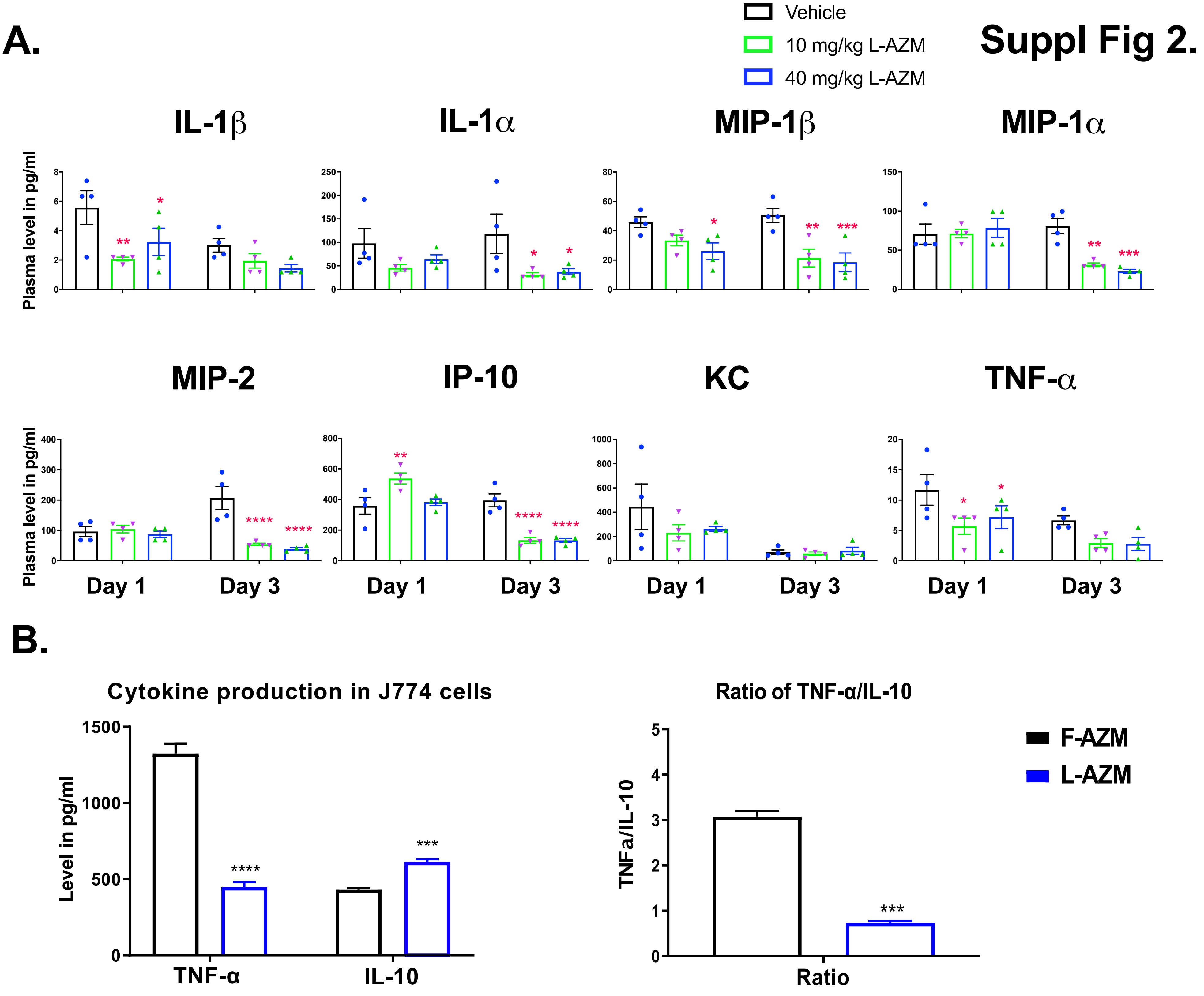

Supplemental Figure 2. L-AZM therapy after MI modulates systemic pro-inflammatory cytokine and chemokine production. (A) Analysis of plasma cytokine levels, conducted using Luminex assay, demonstrates significant reduction in inflammatory cytokine and chemokine levels at days 1 and 3 with L-AZM therapy (n = 4 animals/group/time point, *P<0.05, **P<0.01, ***P<0.001, and ****P<0.0001 compared to the vehicle control). (B) L-AZM produces a more significant shift in macrophage response towards the anti-inflammatory state. Quantitative analyses of pro-inflammatory cytokine, TNF-α, and anti-inflammatory cytokine, IL-10, production from J774 macrophages subjected to LPS stimulation for 48 hours. The analyses demonstrate a more significant reduction of TNF- α with L-AZM compared to F-AZM at 48 hours post-LPS stimulation. Moreover, IL-10 production is additionally enhanced with L-AZM treatment (two independent experiments and 4 replicates/time point, ***P<0.001 and **** P<0.0001 compared to free AZM). Data presented as mean ± SEM. F-AZM, free formulation of azithromycin; L-AZM, liposomal azithromycin; IL-1, interleukin 1; KC, Keratinocyte chemoattractant; IP-10, Inducible protein 10; MIP, macrophage inflammatory protein; TNF-α, tumor necrosis factor-alpha.
